# Osteoporosis treatment in Austria—assessment of FRAX-based intervention thresholds for high and very high fracture risk

**DOI:** 10.1007/s11657-022-01175-w

**Published:** 2022-11-11

**Authors:** Hans P. Dimai, Helena Johansson, Nicholas C. Harvey, Mattias Lorentzon, Enwu Liu, Liesbeth Vandenput, Astrid Fahrleitner-Pammer, Peter Pietschmann, Christian Muschitz, Eugene V. McCloskey, John A. Kanis

**Affiliations:** 1 Division of Endocrinology and Diabetology, Department of Internal Medicine, Medical University of Graz, Graz, Austria; 2 Mary McKillop Institute for Health Research, Australian Catholic University, Melbourne, Australia; 3 Centre for Metabolic Bone Diseases, University of Sheffield Medical School, Beech Hill Road, Sheffield, S10 2RX UK; 4 Sahlgrenska Osteoporosis Centre, Institute of Medicine, University of Gothenburg, Gothenburg, Sweden; 5 MRC Lifecourse Epidemiology Unit, University of Southampton, Southampton, UK; 6 NIHR Southampton Biomedical Research Centre, University of Southampton and University Hospital Southampton NHS Foundation Trust, Southampton, UK; 7 Institute of Pathophysiology and Allergy Research, Center for Pathophysiology, Infectiology and Immunology, Medical University of Vienna, Vienna, Austria; 8 2nd Medical Department, Vinforce, St. Vincent Hospital Vienna, Vienna, Austria; 9 Mellanby Centre for Bone Research, Department of Oncology and Metabolism, University of Sheffield, Sheffield, UK

**Keywords:** Austria, FRAX, Intervention threshold, High risk, Very high risk

## Abstract

***Summary*:**

The adoption of the management pathway proposed by the National Osteoporosis Guideline Group (NOGG), UK applied using the Austrian FRAX® tool in a referral population of Austrian women categorises 22–29% of women age 40 years or more eligible for treatment of whom 28–34% are classified at very high risk.

**Purpose:**

The aim of this study is to provide a reference document for the further development of existing guidelines for the management of osteoporosis in Austria, considering FRAX-based intervention thresholds for high and very high fracture risk.

**Methods:**

The model development was based on two Austrian hospital referral cohorts. Baseline information was collected to compute the 10-year probability (using the Austrian FRAX model) of a major osteoporotic fracture (MOF) and hip fracture both with and without the inclusion of femoral neck bone mineral density (BMD). Assessment thresholds for BMD testing were defined, as well as intervention thresholds. In addition, thresholds that characterise men and women at high and very high fracture risk were established. The management pathway followed that currently recommended by the UK National Osteoporosis Guideline Group (NOGG).

**Results:**

The two cohorts comprised a total of 1306 women and men with a mean age of 66.7 years. Slightly more than 50% were eligible for treatment by virtue of a prior fragility fracture. In those women without a prior fracture, 22% (*n* = 120) were eligible for treatment based on MOF probabilities. Of these, 28% (*n* = 33) were found to be at very high risk. When both MOF and hip fracture probabilities were used to characterise risk, 164 women without a prior fracture were eligible for treatment (29%). Of these, 34% (*n* = 56) were found to be at very high risk. Fewer men without prior fracture were eligible for treatment compared with women.

**Conclusion:**

The management pathway as currently outlined is expected to reduce inequalities in patient management. The characterisation of very high risk may aid in the identification of patients suitable for treatment with osteoanabolic agents.

## Introduction

Osteoporosis is defined as a systemic skeletal disease characterised by low bone mass and microarchitectural deterioration of bone tissue with a consequent increase in bone fragility and susceptibility to fracture [1]. Vertebral fractures, hip fractures and fractures of the proximal humerus and the distal forearm are together considered major osteoporotic fractures (MOF). They have been shown to be associated with an increased risk of subsequent fracture, reduced quality of life, disability and increased mortality [2–4]. The average lifetime risk of a 50-year-old woman to sustain an MOF has been estimated at close to 50%, and at 22% in women and men, respectively [5]. In general, osteoporotic fractures can occur also at many other anatomical sites, such as the pelvis, the tibia or the ribs [6].

The Republic of Austria is located in the southern part of Central Europe. In 2022, the population of Austria was 9.1 million, of whom 3.6 million were age 50 years or more [7]. Similar to other countries of the European Union, its age pyramid shows a narrow base and an increasing proportion of older individuals. The current trend of a growing and ageing population is expected to continue in the coming years and hence be accompanied by an increasing burden of osteoporosis and fragility fracture [8].

The epidemiology of fractures in Austria has been investigated extensively in the past decade. The incidence of hip fracture over the past 3 decades (1989–2018) indicated a steep increase in the first 20 years, which thereafter remained fairly stable over the last decade with a slight albeit significant downward trend [9]. Notwithstanding, hip fracture incidence in Austria has been shown to be among the highest worldwide, exceeded only by Sweden and Denmark [10].

Currently, osteoporosis treatment decisions in Austria are mainly based on country specific guidelines that have been authored and updated in 2017 by an expert group on behalf of the Austrian Bone and Mineral Society, the then Association of Austrian Social Insurance Institutions (Hauptverband der österreichischen Sozialversicherungsträger) and other stakeholders of the Austrian health care system [11]. In general, the recommendation is to initiate osteoporosis specific treatment in people age 50 years or more, with a prior fragility fracture, particularly at the hip or spine. In addition, assessment of 10-year fracture probability by using the country specific version of the fracture-risk assessment tool FRAX® is recommended. Similar to some other countries [12], the Austrian guidelines recommend osteoporosis treatment if the FRAX-based 10-year fracture probability is equal to or is above 20% for MOF, or 5% for hip fracture. The use of fixed intervention thresholds instead of age-specific thresholds raises some anomalies. For example, a person below the age of 65 years might not receive adequate osteoporosis treatment if his or her FRAX-based 10-year fracture probability for MOFs was below 20%, but above the probability of a person of the same age with a prevalent fracture.

Moreover, the treatment rates after osteoporotic fractures in Austria has been shown to be very low [13] despite the availability of national osteoporosis guidelines. Indeed, only 2 out of 10 women, and only 1 out of 10 men receive adequate osteoporosis treatment within 4–18 months after a fracture, irrespective of the fact that Austria’s Gross Domestic Product (GDP) per capita is among the top twenty-five countries worldwide [14] and has a generous social security system which ensures that all receive medical service free of charge.

Against this background, the aim of the present study was to provide a reference document that would help advance country specific osteoporosis guidelines for Austria. In this regard, the Austrian Society of Bone and Mineral Research wished to consider the following. First, the FRAX-based Austrian intervention threshold—which currently is more or less arbitrarily set at a fixed level of 20% for MOFs, and 5% for hip fracture, respectively—should be replaced by hybrid thresholds as recently developed by the UK National Osteoporosis Guideline Group (NOGG), whereby a fixed intervention threshold should be used from the age of 70 years, in order to achieve equity between individuals with versus without a prior fracture at this age [15]. Second, the category of a ‘very high fracture risk’ should be implemented into this hybrid model, as developed by the International Osteoporosis Foundation (IOF) and the European Society for Clinical and Economic Aspects of Osteoporosis and Osteoarthritis (ESCEO) [16], and incorporated into recent NOGG guidance [17].

## Methods

The model development was based on two Austrian hospital referral cohorts (Cohorts A and B). Cohort A was recruited within the framework of the PoCOsteo study, a two-centre prospective study in Austria and Iran, which aimed to develop a point-of-care device for measurement of bone proteomic and genomic markers from a finger-prick whole blood sample [18]. The study population used for model development herein comprised the first consecutive 750 patients age 50 years or above (range 50–89 years) who were referred for skeletal assessment to a large tertiary hospital in the southern region of Austria during 2017–2020 (University Hospital of Graz).Cohort B comprised 89 men and 467 women referred for assessment of osteoporosis to a tertiary hospital in Vienna between 2009 and 2010 and followed up for 5 years or more [19]. This cohort was a pre-planned sample of the total referral population of 2789 patients (502 men and 2278 women). The cohort excluded patients with high-trauma fractures, premenopausal women, patients with malignancies or immobile individuals as well as patients who had previously received specific osteoporosis treatment (except calcium and/or vitamin D).

### Baseline investigation

Baseline information was collected to enable computation of the 10-year probability of a MOF and hip fracture both with and without the inclusion of femoral neck bone mineral density (BMD) with the use of the Austrian FRAX model. BMD was measured with Lunar iDXA (Cohort A) and Lunar Prodigy (Cohort B), from which a standardised BMD was calculated for input into the FRAX model [20]. T-scores were calculated using the NHANES reference values for young Caucasian women as used in FRAX [21].

For Cohort A, age, sex and data on prior fragility fracture and secondary osteoporosis were available in all men and women. Body mass index (BMI) and BMD were missing in 12 and 34 patients, respectively (1.6% and 4.5%, respectively). With regard to the other dichotomous FRAX variables, information was incomplete for parental history of hip fracture (*n* missing = 49, 6.5%), exposure to glucocorticoids (*n* = 4, 0.5%), rheumatoid arthritis (*n* = 3, 0.4%), current smoking (*n* = 3, 0.4%) and high alcohol consumption (*n* = 3, 0.4%). For the purposes of this analysis, these variables were simulated to more closely represent the population from which the sample was drawn. In Cohort B, age, sex and body mass index (BMI) were available in all men and women. BMD was missing in 20 patients (3.6%). With regard to the other dichotomous FRAX variables, information was incomplete for prior fracture (*n* missing = 107, 19.2%)), parental history of hip fracture (*n* = 340, 61.2%)), exposure to glucocorticoids (*n* = 104, 18.7%), rheumatoid arthritis (*n* = 83, 14.9%), current smoking (*n* = 180, 32.4%), secondary osteoporosis (n = 105, 18.9%) and high alcohol consumption (*n* = 181, 32.6%). For the purposes of this analysis, these variables were simulated.

### Simulation of risk variables

An analysis was performed where the missing values on clinical risk factors were simulated based on the cohort itself using associations between dichotomous FRAX variables to generate logistic regression equations as described previously [22, 23]. The equations were applied to the data in the present sample to predict the probability of having a positive value for the missing key risk factor for each individual. Next, a random number between 0 and 1 was generated using a computer program, which was then compared with the predicted probability for that variable for that individual. If the random number was less than or equal to the predicted probability, the individual was assigned a positive response for the risk factor. If the random number was larger than the predicted probability, the person was assigned a negative response for the risk factor. The simulations for femoral neck BMD and BMI were based on examining the conditional probability of the association of the risk factors. Probabilities in the cohorts following simulation did not differ from the subsets that had all variables (see Appendix).

### Fracture probabilities

The 10-year probabilities of hip fracture and a major osteoporotic fracture (clinical spine, hip, humerus or distal forearm fracture) were calculated using the FRAX model for Austria (web version 4.1). Calculations were undertaken with and without the inclusion of femoral neck BMD.

### Intervention thresholds

**Fig. 1:**
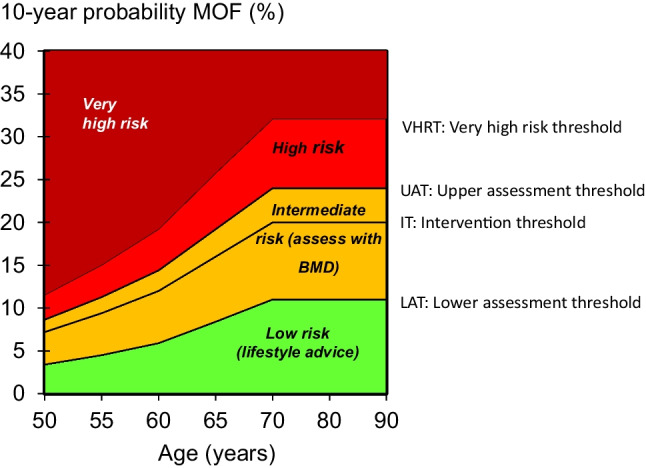
Assessment, intervention and risk thresholds for major osteoporotic fracture probability (MOF) in Austria with the use of FRAX. Individuals with probabilities below the lower assessment threshold (LAT) are considered for lifestyle advice. Those at intermediate risk (probabilities between the upper assessment threshold (UAT) and lower assessment threshold (LAT)) are further assessed with BMD measurement. Where probabilities calculated using BMD lie above or below the intervention threshold (IT), treatment or lifestyle advice, respectively, is recommended. Patients with probabilities above the upper assessment threshold (UAT) are considered for treatment. Those with probabilities above the very high-risk threshold (VHRT) might be considered for osteoanabolic therapy if clinically appropriate. Where BMD measurement is not practical, patients with probabilities above the IT are considered for treatment

The use of FRAX in clinical practice demands a consideration of the fracture probability at which to intervene, both for treatment (an intervention threshold) and for BMD testing (assessment thresholds). The approach to the setting of intervention and assessment thresholds used the methodology adopted by NOGG for FRAX-based guidelines in the UK [24–26]. For men and women, the intervention threshold up to age 70 years is set at a risk equivalent to that of a woman of the same age with a prior fracture and therefore rises with age. At age 70 years and above, fixed thresholds are applied [15]. A threshold that characterises men and women at high and very high fracture risk has also been established; very high risk is identified as a FRAX-based fracture probability that exceeds the intervention threshold by 60% (Fig. 1) [17].

Numerical values for thresholds are given in Table 1.

**Table 1:** Numerical values for thresholds for major osteoporotic fracture and for hip fracture probabilities (% over 10 years) based on the Austrian version of FRAX

	Major osteoporotic fracture					Hip fracture	
Age (years)	LAT	UAT	IT	VHRT		IT	VHRT
40	2.9	7.8	6.5	10.3		0.6	1.0
45	3.0	8.0	6.7	10.6		0.9	1.5
50	4.2	10.9	9.1	14.6		1.4	2.2
55	5.3	13.3	11.1	17.7		1.9	3.1
60	6.1	15.0	12.5	20.0		2.7	4.4
65	8.5	19.9	16.6	26.5		4.3	6.9
70	11.6	25.6	21.3	34.1		7.1	11.3

*LAT* lower assessment threshold, *UAT* upper assessment threshold, *IT* intervention threshold, *VHRT* very high-risk threshold

### Assessment thresholds

Two assessment thresholds for making recommendations for the measurement of BMD were considered [24, 25]:

- A threshold probability below which neither treatment nor a BMD test should be considered (lower assessment threshold, LAT).
- A threshold probability above which treatment may be recommended irrespective of BMD (upper assessment threshold, UAT).

The LAT was set to exclude a requirement for BMD testing in men and women without clinical risk factors, as given in current European guidelines [27–29]. It was therefore set to the age-specific 10-year probability of a major fracture equivalent to women with no clinical risk factors. The UAT was chosen to minimise the probability that a patient, characterised to be at high risk using clinical risk factors alone, would be reclassified to be at low risk with additional information on BMD and vice versa [30]. The upper assessment threshold was set at 1.2 times the intervention threshold as used in the UK (see Table 1) [25].

### Management pathway

The management pathway followed that currently recommended by NOGG [31]. Under the NOGG strategy, the risk of fracture is first assessed on clinical risk factors alone which in turn provides guidance whether a femoral neck BMD measurement or treatment is indicated, an approach that has been endorsed by the UK National Institute for Health and Care Excellence [32]. An exception is in the presence of a prior fragility fracture, in which case treatment is to be considered in such patients without necessarily undertaking a BMD measurement. For the present report, we assumed that treatment would be considered in all men and women with prior fracture. In those with clinical risk factors (but no prior fracture), the decision is based on the 10-year probability of major osteoporotic fracture with some individuals deemed at high risk or very high risk (treatment without BMD), some at or near the intervention threshold (intermediate risk; BMD indicated to finalise risk evaluation and stratification) and some at low risk (lifestyle advice, reassurance and re-evaluation in the future). Once BMD is entered into the calculation, the decision to treat or not is based on a comparison to age-specific thresholds for major osteoporotic fracture probability; a probability at or above the intervention threshold indicates eligibility for treatment.

The NOGG guidance also recommends, when BMD is included in a FRAX assessment, that hip fracture probability can be additionally taken into account and that high risk and very high risk be predicated the higher of the two (MOF and hip fracture) risk assessments. The impact of this additional assessment was also explored.

### Prior assessment strategy

The previous Austrian guideline recommends osteoporosis specific treatment in individuals age 50 years or more, with a prior fragility fracture. In addition, it recommends osteoporosis treatment if the FRAX-based 10-year fracture probability is ≥ 20% for MOF, or ≥ 5% for hip fracture. Since patients with a prior fracture would be treated by either approach, we compared risk categorisation of the previous guideline (prior assessment strategy) with that investigated in the present study based on the 10-year probability of MOF with BMD included (NOGG assessment strategy). Concordance was assessed using Cohen’s kappa.

### Sensitivity analysis

The Austrian Bone and Mineral Society have raised the view that in Austria no patient should be assessed for 10-year fracture probability without also measuring BMD. In this scenario, there would be no need for an ‘intermediate’ risk based on which BMD could be performed in order to stratify patients to either above or below the lower intervention threshold. The impact of this on the management pathway was explored as a sensitivity analysis.

## Results

**Fig. 2:**
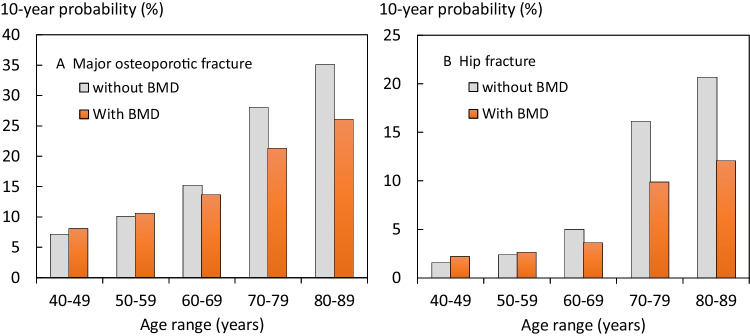
Mean 10-year probabilities of **A**) a major osteoporotic fracture and **B**) hip fracture by age with and without the inclusion of BMD, in pooled cohorts

The baseline characteristics are given in Table 2. The prevalence of the clinical risk factors was high as would be expected from referral populations. Fracture probabilities rose with age (Fig. 2). For both MOF and hip fracture probabilities, the inclusion of BMD in the calculation was associated with a decrease in fracture probability in men and women age 60 or more years. The effect was particularly marked for hip fracture probability.

**Table 2:** Summary description of the baseline variables in the pooled referral cohorts (including simulated data). The last column gives the proportion of data simulated for each variable

	*N*	Mean	SD	%	% simulated
Age (years)	1306	66.7	10.3		0
BMI (kg/m^2^)	1306	25.2	4.7		0.9
Femoral neck BMD (*T*-score)	1306	− 1.63	0.89		4.1
Female	1116			85.5	0
Previous fracture	664			50.8	8.2
Current smoking	170			13.0	14.0
Secondary osteoporosis	211			16.2	8.0
Alcohol 3 or more units per day	62			4.7	14.1
Parental history of hip fracture	172			13.2	29.8
Glucocorticoid exposure	148			11.3	8.3
Rheumatoid arthritis	85			6.5	6.6
Ten-year probability		Median	IQR	Range	
Hip fracture probability calculated without BMD	1306	4.97	2.06–11.71	0.1–76.1	
Hip fracture probability calculated with BMD	1306	3.47	1.45–7.44	0.0–65.0	
MOF probability calculated without BMD	1306	15.80	8.90–26.12	2.2–79.8	
MOF probability calculated with BMD	1306	13.80	8.40–20.79	2.5–70.4

### Management pathway

**Fig. 3:**
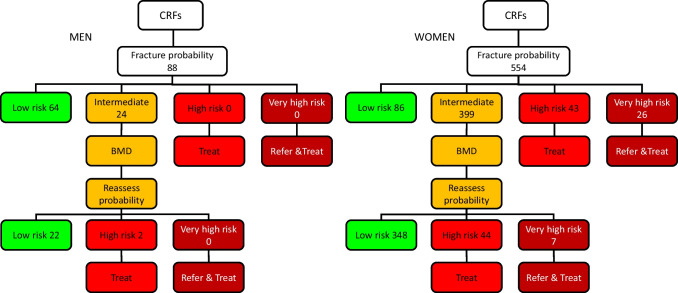
Assessment pathway and categorisation of risk in men and women without a prior fracture

A prior fragility fracture was recorded in 562 of 1116 women (50.4%) and 102 of 190 men (53.7%), and these patients would be eligible for treatment on this basis. For those without a prior fracture (554 women and 88 men), the assessment of fracture probability and the categorisation of fracture risk is undertaken on the basis of age, sex, BMI and the clinical risk factors. In women, 69 (6.2%) would be eligible for treatment in that their fracture probability for MOF exceeded the intervention threshold for Austria (Fig. 3). Of these, 26 (2.3%) were categorised at very high risk. At the other extreme, 86 women were categorised as low risk individuals (7.7%) and would not normally be eligible for further assessment in that their fracture probability lay below the lower assessment threshold. The intermediate category of risk in Fig. 3 comprised 399 women (35.8%) in whom FRAX would be recalculated with the inclusion of femoral neck BMD. Of these, 348 women were categorised at low risk (31.2%) and 44 women at high risk (3.9%) and 7 women at very high risk (0.6%). In brief, of 554 women with no prior fracture, 22% (*n* = 120) were eligible for treatment. Of these, 28% (*n* = 33) were found to be at very high risk.

Where eligibility for treatment was based on hip fracture probabilities (in addition to MOF probabilities), 164 women without a prior fracture were eligible for treatment. Of these, 38% (*n* = 3) were found to be at very high risk.

In men with no prior fracture, the initial assessment (FRAX without BMD) categorised 34% at low risk and 13% at intermediate risk. Of those at intermediate risk, all but two male patients (1%) fell into the low-risk category when assessed using MOF probabilities. Where eligibility for treatment was based on hip fracture probabilities (in addition to MOF probabilities), 8 men without a prior fracture were eligible for treatment. Of these, 34% (*n* = 56) were found to be at very high risk.

The overall disposition of the cohort is summarised in Table 3.

**Table 3:** Disposition of cohort. Ten-year probabilities of a major osteoporotic fracture (MOF) are calculated with BMD

		Men			Women		
		*n*	%	MOF probability*	*n*	%	MOF probability*
A	All	190	100	10.3	1116	100	17.0
B	Prior fracture	102	53.7	13.3	562	50.4	21.8
C	No prior fracture	88	46.3	6.9	554	49.6	12.1
D**	Low risk	86	45.3	6.8	434	38.9	9.3
E**	High risk	2	1.1	14.5	87	7.8	19.6
F**	Very high risk	0	0.0	-	33	3.0	29.3
G	Treated (MOF)	104	54.7	13.3	682	61.1	21.8
	BMD tests	24	12.6	9.8	399	35.8	11.2
MOF + HF							
H**	Low risk	80	42.1	6.4	390	34.9	8.7
I**	High risk	5	2.6	10.2	108	9.7	17.8
J**	Very high risk	3	1.6	15.2	56	5.0	24.5

*Calculated with BMD.

**In those without prior fracture history

### Prior fracture

A total of 102 men and 562 women had a history of a prior fracture and would thus be eligible for treatment without necessarily having a BMD test. If a BMD were to be undertaken and the probability of a MOF calculated, then 12 men and 117 women (12% and 21% of men and women with a prior fracture, respectively) would be categorised at very high risk. If hip fracture probability were additionally evaluated, then 27 men and 215 women would be categorised at very high risk (26% of men and 38% of women with a prior fracture).

### Sensitivity analysis

The impact of undertaking BMD measurements in all patients alongside FRAX is summarised in Table 4 for individuals without a prior fracture. In the case of women, 458 of 554 (83%) would be characterised at low risk, 76 (14%) at high risk and 20 (4%) at very high risk. These data can be compared with the classic NOGG strategy summarised in Fig. 3 and Table 3. Of 554 women with no prior fracture, the number in the low risk category decreased by 24 with the universal use of BMD. Conversely, the number of high risk women increased by 11 and very high risk women by 13. As expected, if hip fracture probability were additionally evaluated, then an additional 6 men and 4 women would be categorised at high risk and an additional 7 men and 7 women would be categorised at very high risk.

**Table 4:** Disposition of cohort when BMD is entered into FRAX® in all patients (with no prior fragility fracture). Categorisation of risk was determined from probabilities of a major osteoporotic fracture (MOF) or the combination of hip fracture (HF) and MOF probabilities

		Men			Women		
		*N*	%	MOF probability with BMD	*N*	%	MOF probability with BMD
MOF	Category	88	100.0	6.9	554	100.0	12.1
	Low risk	84	95.5	6.5	458	82.7	9.7
	High risk	3	3.4	14.0	76	13.7	21.1
	Very high risk	1	1.1	18.6	20	3.6	33.5
MOF or HF	Low risk	73	83.0	6.1	402	72.6	9.0
	High risk	8	9.1	8.4	91	16.4	17.2
	Very high risk	7	8.0	13.5	61	11.0	25.1

When both MOF and hip fracture probability were used to characterise risk the ‘BMD in all’ strategy identified 9.1% of men at high risk and 8.0% at very high risk. With the NOGG strategy, the respective proportions were lower (5.7% and 3.4%, respectively). In women, the ‘BMD in all’ strategy identified 16.4% at high risk and 11.0% at very high risk. With the NOGG strategy, the respective proportions were lower (19.5% and 10.1%, respectively).

A principal difference in the two strategies lies in the requirement for BMD testing. Testing all patients (without prior fragility fracture) with a BMD measurement and using MOF intervention thresholds require 6.4 scans to identify one patient for treatment, whereas the NOGG strategy requires 3.5 scans to identify one patient for treatment.

The impact of testing BMD in all patients (with or without a prior fracture) is given in Table 5. The number of individuals identified at high or very high risk varied according to the criteria used to characterise high and very high risk. When assessed from the 10-year probability of MOF, 15.8% of men and 26.5% of women were at high risk and 6.3% and 22.9%, respectively, at very high risk (Table 5). The proportion of individuals characterised at very high risk increased to 13.7 and 28.1% in men and women, respectively, when additionally based on hip fracture probability.

**Table 5:** Number and proportion (%) of the referral cohort characterised at high and very high risk based on 10-year probabilities of a major osteoporotic fracture (MOF) and on 10-year probabilities of MOF or hip fracture (HF). Results combine data for those with and without a prior history of fracture

Probability criterion	Sample size		High risk (%)		Very high risk (%)	
	Men *n*	Women *n*	Men	Women	Men	Women
MOF	190	1116	30 (15.8)	296 (26.5)	12 (6.3)	256 (22.9)
MOF or HF	190	1116	42 (22.1)	285 (25.6)	26 (13.7)	314 (28.1)

### Comparison with prior assessment strategy

In those without a fragility fracture, the number of individuals eligible for treatment identified using the NOGG approach was fewer than that using the prior assessment strategy. For example, using the NOGG approach based on MOF probability, 19.0% of the cohort were identified for treatment, whereas 25.4% were identified by the prior Austrian guidance. As would be expected, there were differences in the individuals identified using the two assessment algorithms. Cohen’s Kappa was 0.574 signifying only moderate concordance in the categorisation of patients. The distribution of concordant and discordant classification is shown in Table 6. As would be expected, 10-year MOF probabilities were high in the 95 individuals characterised as eligible for treatment using both algorithms (24.3%), low in 452 individuals characterised at low risk by both algorithms (7.7%) and intermediate where characterisation was discordant (14.6 an 16.5%). The major difference between the two approaches was in the age of those identified at high risk. With the prior guideline approach, the mean age of those eligible for treatment was 73.9 years, whereas with the NOGG strategy in the present study included more younger individuals (mean age 68.9 years).

**Table 6:** Categorisation and characteristics of men and women with the prior assessment strategy or NOGG assessment strategy

NOGG assessment	Prior assessment	*n*	Women (%)	Age (years)	FRAX MOF (%)*
Treatment	Treatment	95	99	73.8	24.3
No treatment	No treatment	452	82	62.4	7.7
Treatment	No treatment	27	96	63.9	14.6
No treatment	Treatment	68	91	74.0	16.5

*FRAX MOF (%), 10-year probability of MOF with BMD

## Discussion

The aim of the present study was to provide a reference document that would help advance existing country specific osteoporosis guidelines for Austria, considering a management pathway following that currently recommended by the National Osteoporosis Guideline Group (UK). In particular, the Austrian Bone and Mineral Society defined three aspects that should be integrated into the existing guidelines. First, the currently more or less arbitrarily fixed intervention threshold based on FRAX probabilities should be replaced by age-specific thresholds up to the age of 70 years. Second, a fixed intervention threshold should be used from the age of seventy. And third, the category of a ‘very high risk’, as recommended recently by the IOF and the ESCEO, should be implemented into this hybrid model.

The categorisation of risk is widely accepted within medicine as an appropriate mechanism to direct decisions on treatment; examples include the fields of cardiovascular disease, hypertension and diabetes [33–35].The further sub-categorisation of those meriting treatment into high risk and very high risk is predicated on the same principle as it aids in choosing the type of treatment to be recommended. The increasing availability of anabolic therapies in osteoporosis and their superiority to anti-resorptive treatments in head-to-head randomised clinical trials has influenced discussions about the setting of threshold values [36–39]. Such considerations justify the need for dichotomy but are less helpful in its operationalisation, which by nature will always be somewhat arbitrary. With regard to the development of thresholds between high and very high fracture risk NOGG, ESCEO and the IOF identify approximately 10% at very high risk with a risk threshold consistent with patients included in phase 3 studies of anabolic agents considered precedent and appropriate [16, 17].

The present study examines the impact of categorising fracture risk in two referral cohorts using the methodology developed by NOGG but applied to the Austrian FRAX model. Fracture probabilities were higher than those which would be expected in the general population because of the high frequency of clinical risk factors consistent with a referral population. It is of interest that for both MOF and hip fracture probabilities the inclusion of BMD in the FRAX calculation was associated with a decrease in fracture probability at older ages. This suggests a referral bias towards excluding individuals with low BMD, perhaps because of immobility, institutionalized residence or multiple comorbidities.

The increase in the numbers in the very high risk category when both the MOF and hip fracture thresholds are used needs consideration in guideline development. A further option examined was to undertake BMD testing in all patients, rather than in those in the ‘intermediate’ category of risk. This increased modestly the proportion of individuals detected at very high risk using the MOF intervention thresholds but had a more marked effect when hip fracture and MOF thresholds were applied. As noted in the results, testing all patients (without prior fragility fracture) with a BMD measurement and using MOF intervention thresholds required 6.4 scans to identify one patient for treatment whereas the NOGG strategy required 3.5 scans to identify one patient for treatment. These differences also need consideration in guideline development.

This analysis has a number of strengths and limitations. A potential consideration is that some of the risk factor information needed was missing in the cohorts and had to be simulated. The weakness of simulation is that there is a loss of accuracy for those individuals in whom missing variables were simulated. However, this is less relevant for populations, and summary data more closely reflect the sample from which data were drawn with a benefit of optimising sample size. In this context, we checked whether probabilities in the two cohorts studied following simulation differed from the subsets that had all variables. There was no difference in fracture probabilities indicating the adequacy of the simulation. The present analysis was conducted in two referral cohorts and does not necessarily represent the impact of categorisation in the entire population at risk. Moreover, there was evidence of some referral bias. Notwithstanding, the present assessment is not inconsistent with findings from a population-based simulated cohort in the UK [17]. Also, the cohorts were drawn from a different decade, though there was little difference in baseline characteristics between the two cohorts. It should be noted that our analysis could not take into account the recency of a prior fracture. Were this to be taken into account, then the prevalence of very high risk would be expected to increase. There are no empirical data to calculate the quantum of effect but has been indirectly estimated to categorise an additional 2.9% of women age 50 years or more [24].

Finally, it will be important to place the upper intervention thresholds in a health economic perspective. In the context of osteoporosis and fracture risk, the intervention threshold that is relevant for payers can be defined as the probability of fracture at which intervention becomes cost-effective. Whilst NOGG thresholds are driven by clinical appropriateness rather than health-economics, it is still important to underpin the chosen intervention thresholds by cost-effectiveness [40]. The lower intervention threshold (LIT) used in the NOGG guidance provides strategies that are highly cost-effective [26]. The upper intervention thresholds examined in this report require health economic validation using models that can accommodate the heightened risk associated with the recency of fracture [41].

In summary, the present study provides a reference document for further development and updating of country specific osteoporosis guidelines in Austria. It considers the recently recommended category of ‘very high fracture risk’, as well as age-dependent intervention thresholds up to an age of 70 years, and a fixed intervention threshold thereafter. The implementation of an improved case-finding strategy together with less arbitrary but more scientifically driven age-specific FRAX-based intervention thresholds will help improve management of osteoporosis patients in Austria and reduce the marked osteoporosis treatment gap in this country. Furthermore, the characterisation of very high risk may aid in the identification of patients suitable for treatment with osteoanabolic agents.

## Appendix

Table 7, Table 8

**Table 7:** Summary description of the baseline variables in cohort A. Baseline variables and fracture probabilities in the cohort with simulation did not differ from the subset of 656 individuals that had all variables

	Original cohort (those with FRAX without BMD)				Original cohort plus simulated variables			
	*N*	Mean	SD	(%)	*N*	Mean	SD	(%)
Age (years)	656	64.6	8.5		750	64.9	8.5	
BMI (kg/m^2^)	656	24.8	4.5		750	24.9	4.5	
Femoral neck BMD (*T*-score)	656	− 1.56	0.84		750	− 1.56	0.85	
Female	570			86.9	649			86.5
Previous fracture	325			49.5	386			51.5
Current smoking	66			10.1	80			10.7
Secondary osteoporosis	115			17.5	135			18.0
Alcohol 3 or more units per day	10			1.5	17			2.3
Parental history of hip fracture	102			15.5	117			15.6
Glucocorticoid exposure	99			15.1	117			15.6
Rheumatoid arthritis	47			7.2	57			7.6
Ten-year probability								
Hip fracture probability calculated without BMD	656	7.65	9.58		750	7.96	9.81	
Hip fracture probability calculated with BMD	656	4.77	6.25		750	4.98	6.47	
MOF probability calculated without BMD	656	17.91	12.46		750	18.38	12.74	
MOF probability calculated with BMD	656	14.84	9.20		750	15.21	9.56	

**Table 8:** Summary description of the baseline variables in cohort B. Baseline variables and fracture probabilities in the cohort with simulation did not differ from the subset of 132 individuals that had all variables

	Original cohort (those with FRAX without BMD)				Original cohort plus simulated variables			
	*N*	Mean	SD	(%)	*N*	Mean	SD	(%)
Age (years)	132	67.4	10.8		556	69.1	11.9	
BMI (kg/m^2^)	132	26.1	5.9		556	25.6	4.9	
Femoral neck BMD (*T*-score)	132	− 1.83	0.83		556	− 1.73	0.94	
Female	107			81.1	494			84.0
Previous fracture	71			53.8	290			50.0
Current smoking	29			22.0	102			16.2
Secondary osteoporosis	20			15.2	85			13.7
Alcohol 3 or more units per day	3			2.3	53			8.1
Parental history of hip fracture	18			13.6	58			9.9
Glucocorticoid exposure	6			4.5	34			5.6
Rheumatoid arthritis	6			4.5	30			5.0
Ten-year probability								
Hip fracture probability calculated without BMD	132	8.3	8.2		556	9.8	10.8	
Hip fracture probability calculated with BMD	132	6.8	7.2		556	7.0	8.4	
MOF probability calculated without BMD	132	18.8	12.0		556	20.1	13.6	
MOF probability calculated with BMD	132	17.2	10.3		556	17.1	11.2	
